# Detection of Induced GNSS Spoofing Using S-Curve-Bias

**DOI:** 10.3390/s19040922

**Published:** 2019-02-22

**Authors:** Wenyi Wang, Na Li, Renbiao Wu, Pau Closas

**Affiliations:** 1 Tianjin Key Lab for Advanced Signal Processing, Civil Aviation University of China, Tianjin 300300, China; nali_nasy@163.com (N.L.); rbwu@vip.163.com (R.W.); 2 Electrical & Computer Engineering Department, Northeastern University, 360 Huntington Avenue, Boston, MA 02115, USA; closas@northeastern.edu

**Keywords:** global navigation satellite system (GNSS), induced spoofing, S-curve-bias (SCB), Texas spoofing test battery (TEXBAT)

## Abstract

In Global Navigation Satellite System (GNSS), a spoofing attack consists of forged signals which possibly cause the attacked receivers to deduce a false position, a false clock, or both. In contrast to simplistic spoofing, the induced spoofing captures the victim tracking loops by gradually adjusting it’s parameters, e.g., code phase and power. Then the victims smoothly deviates from the correct position or timing. Therefore, it is more difficult to detect the induced spoofing than the simplistic one. In this paper, by utilizing the dynamic nature of such gradual adjustment process, an induced spoofing detection method is proposed based on the S-curve-bias (SCB). Firstly, SCB in the inducing process is theoretically derived. Then, in order to detect the induced spoofing, a detection metric is defined. After that, a series of experiments using the Texas spoofing test battery (TEXBAT) are performed to demonstrate the effectiveness of the proposed algorithm.

## 1. Introduction

Global navigation satellite system (GNSS) is a general term for various satellite-based navigation systems and their augmentation systems. The application of GNSS is omnipresent, including mobile phone location, the smart grid, emergency rescue, fishing operation, precision guidance and strike of weapons, the transport and management of air, sea and ground and so on [1]. In addition, a great deal of new applications based on GNSS are constantly emerging. Taking into account the significance of GNSS applications, its security becomes a pressing issue [2]. GNSS signals broadcasted by the constellations arrive at the antenna with an extremely low signal power level, e.g., approximately 20 dB lower than the noise. Therefore, it is highly susceptible to various types of interferences. As a special form of interference, spoofing does great harm to GNSS. Recent successful implementations of spoofing tests have further enhanced the awareness of the harm of spoofing attacks [3,4]. Spoofers utilize the open transparency and predictability of GNSS civilian signals to generate spoofing signals which have similar signal structure as authentic ones. Thus they can induce a victim receiver to believe that they are authentic signals and provide incorrect navigation messages or incorrect pseudo-ranges to forge a localition solution at the receiver. It is hard for a conventional GNSS receiver to detect the spoofing attack, which may lead to incorrect position or timing information.

There is a rich literature studying this issue. A representative team comes from the radionavigation laboratory in the University of Texas at Austin [5,6,7,8]. According to references [9,10], the type of spoofing is classified as simplistic, intermediate, and sophisticated, depending on their complexity and the level of robustness required to the related anti-spoofing techniques. A simplistic spoofing basically consists of a GNSS signal generator that emits signals which are visually inconsistent (in frequency, phase, code, and data message) with authentic satellite signals. For the simplistic spoofing, in order to successfully attack the victim receivers, it usually needs to first use jamming to unlock the receiver. Then the victim receivers will lock on the forced signal in the process of recapture. The intermediate and sophisticated spoofing adds synchronization blocks, which makes the counterfeit signals consistent with the real ones and result in spoofing attacks which are more difficult to detect. Furthermore, the sophisticated spoofing attack can be accomplished by using multiple transmitting antennas [11], in which case different forged satellite signals can come from different directions. The design and implementation of a multiple-antenna spoofing device is not simple and may seem laborious.

**Figure 1:**
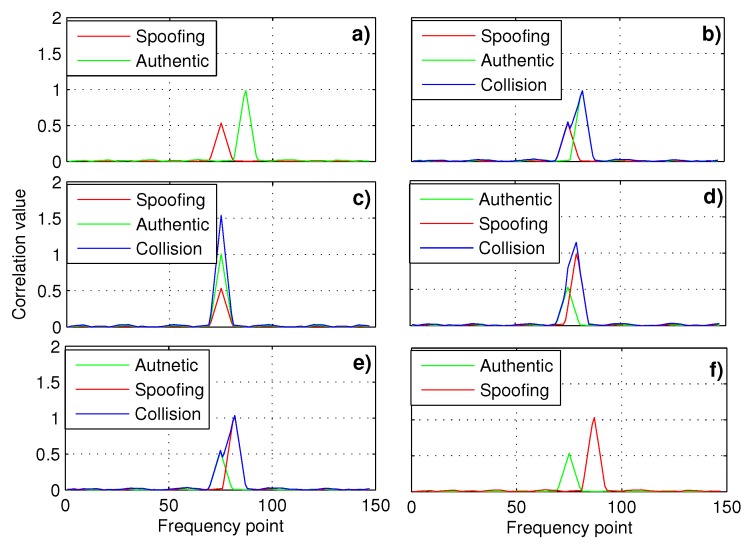
Schematic diagram of the induced spoofing attack process. In (**a**), the spoofing lags the authentic signal with a lower power. From (**a**) to (**c**), the spoofing gradually approaches the authentic signal. Subsequently, the power of spoofing is greater than that of the authentic signal and the receiver is gradually controlled by the spoofing in (**d**–**f**).

In this paper, we focus on the induced spoofing, e.g., GPS L1 C/A signals, where the counterfeit signals are consistent with the real ones, but transmitted with a single antenna. Thus the induced spoofing belongs to intermediate spoofing [12]. The induced spoofing captures the victim tracking loops by gradually adjusting it’s parameters, e.g., code phase and power. Then the victims smoothly deviate from the correct position or timing [13,14]. Therefore, for the conventional receiver, this kind of spoofing is more subtle and will not lead to an unlock in the tracking loop. Figure 1 shows the correlation peak superposition process of the tracking loop correlator in a victim receiver. Figure 1a is the beginning of adjustment process, the spoofing lags the authentic signal by two chips with a lower power and the same frequency, but a different code rate. From Figure 1a–c, the spoofing gradually approaches the authentic signal in code phase. At the same time, the power is gradually increased but is still lower than that of the authentic signal until it is synchronized with the authentic signal in code phase and carrier frequency. Subsequently, the spoofing slowly increases the power beyond that of the authentic signal, pulls off the authentic signal towards right until the receiver is completely controlled by the spoofing as shown in Figure 1d–f. In Figure 1f, it is about two chips code phase ahead of the authentic signal. In fact, the spoofing only needs a slightly higher power to assure a successful locking of the victim receiver. Since the tracking loop maintains lock in this spoofing process, it would lead to an incorrect position or timing for the victim receiver, while no loss of lock will be detected by a conventional GNSS receiver.

The existing anti-spoofing methods can be roughly divided into two categories, namely, detection techniques and suppression techniques. Among them, the detection techniques are designed to identify whether the receiver has been subjected to a spoofing attack, and the purpose of suppression techniques is to help the victim receiver restore its positioning and navigation capability. Here, the existing spoofing detection techniques are briefly reviewed, including spatial-based detection techniques, signal power detection techniques, navigation information detection techniques, integrated navigation detection techniques, encryption authentication techniques, and signal quality monitoring techniques.

Spatially-based detection techniques utilize the characteristics of the spoofer, which transmits multiple satellite signals with the same antenna. On the contrary, the authentic satellite signals come from different directions. Therefore, the spoofing signals are spatially coherent and the spoofing can be identified by determining the correlation degree among satellite signals through a spatial processing technique [15,16,17]. Such techniques are typically costly to implement, since they require the use of multi-antenna receivers and large observation intervals.

For signal power detection techniques, the receivers continuously monitor the power related parameters and declare a spoofing attack when there is an outlier. The parameters related to power include carrier-to-noise ratio [18,19], absolute power, and distribution checks of correlator outputs [20]. These techniques require the receiver to have a high accuracy in measuring parameters of the received signal, and the hardware complexity of the receiver will also increase correspondingly.

Navigation information detection techniques detect signal code rate and phase rate. For the real satellite signals, the Doppler frequency and code rate are generated by the relative motion between the GNSS satellites and the receiver, thus the two have consistency [21,22]. Simple spoofing can not keep the consistency of Doppler frequency and code rate. When there is inconsistency between them, it is decided to suffer from a spoofing attack. This method is invalid for intermediate and sophisticated spoofing attacks because they overcome such consistency checks.

Integrated navigation detection techniques involve combinations of GNSS signals and other navigation devices, which assist the receiver to identify spoofing effectively [23]. This techniques increase the complexity of GNSS receiver’s hardware and software.

Most encryption authentication techniques need to change the structure of GNSS signals and this techniques can not be applied in a short time [24,25,26]. Signal quality monitoring techniques determine whether there is interference by monitoring the correlation distortion of the tracking loop according to the pseudo-code’s auto-correlation property [7,24,26] or even pre-correlation signal quality [27,28]. Several metrics are also proposed in literature [29,30,31]. These techniques, originally designed for multipath detection [32], were recently found to be useful to identify the deformation on the correlation function due to an intermediate spoofing attack. They generally have simple structures with low complexity, showing good feasibility. As in [33,34,35,36], the ratio test metric is used as a measurement compared with predefined thresholds to judge the correlation distortion during the spoofing signals hauling process. However, when the signal’s intervals are large, the two signals are not overlapping, and need to wait until distortion occurs. This means that the method is simple but has a large detection time.

In the field of GNSS signal quality evaluation, there are a series of evaluation parameters for judging whether the signal quality is abnormal. Signal correlation domain analysis indexes have a correlation curve, correlation loss, S-curve-bias (SCB), etc. [37]. SCB is an index that can be used to describe the correlation distortion. Specially, SCB is used to describe the deviation between the highest peak and the symmetric point of the correlation curve. The code loop discriminator curve usually locks in the place where the phase is biased, causing SCB. The bias generally results from the influence of the channel transmission distortion and the nonlinear effects of power amplifiers or multipaths. The correlation peak of an induced spoofing attack is similar to such an abnormal signal. Thus SCB has the potential ability to detect spoofing.

In this paper, by utilizing the dynamic feature of gradual adjustment process, an induced spoofing detection method is proposed based on SCB. The main work of this paper is briefly described as follows. In order to obtain the changing process of SCB, it is derived theoretically for induced spoofing. The theoretical results reflect the whole changing process of induced spoofing. It should be noted that multipath signals can also result in the SCB changing. However, the changing process of multipath is not generally similar with that of induced spoofing. From this point of view, the proposed algorithm is not sensitive to multipath signals in most cases. In addition, front-end filter of receiver may lead to significant SCB variations. However, since only dynamic changes in the SCB are relevant for our method, the the proposed algorithm is also robust to SCB due to the front-end filter. Then a metric is defined to detect induced spoofing. The performance of the proposed algorithm is evaluated with the Texas spoofing test battery (TEXBAT) [38], which has been used to evaluate performance of spoofing detection methods in many papers. In addition, the proposed algorithm is compared with another algorithm based on the ratio test metric, which utilizes the track loop’s correlation distortion.

The paper is organized as follows. The signal model is presented in Section 2. The proposed algorithm is derived in Section 3. Section 4 provides experimental results with the TEXBAT data sets. Conclusions are drawn in Section 5.

## 2. Signal Model

For most GNSS receivers, the received radio frequency (RF) signals will be converted to intermediate frequency (IF) signals. Subsequent processing will be based on the IF signals. When there is an induced spoofing, for a given receiver with single antenna, the received IF signal can be denoted as:

(1) $$x\left(t\right)=x_{a}\left(t\right)+x_{s}\left(t\right)+x_{n}\left(t\right),$$

where $x\left(t\right)$ is the received IF signal, *t* is time in seconds, $x_{a}\left(t\right)$ and $x_{s}\left(t\right)$ are separately authentic satellite signal and spoofing, $x_{n}\left(t\right)$ denotes the additive white Gaussian noise (AWGN) with zero mean and variance $\sigma^{2}$.

The authentic satellite signal is modeled as:

(2) $$x_{a}\left(t\right)=\sum_{i=1}^{M}\sqrt{P_{i}^{a}}D_{i}^{a}\left(t-\tau_{i}^{a}\right)C_{i}\left(t-\tau_{i}^{a}\right)\cos\left(2\pi \left(f_{0}+f_{d,i}^{a}\right)t+\phi_{i}^{a}\right),$$

where *M* is the number of authentic satellites included in the received signal, $P_{i}^{a}$ is the received power of the *i*-th satellite, and $D_{i}^{a}\left(t\right)$ is the ±1-valued *i*-th signal’s data bit stream, $C_{i}\left(t\right)$ is its ±1-valued spreading code, $\tau_{i}^{a}$ is the *i*-th signal’s code phase, $f_{0}$ is the intermediate frequency, $f_{d,i}^{a}$ denotes the Doppler shift of the *i*-th authentic satellite signal in Hertz, and $\phi_{i}^{a}$ is its initial carrier phase.

It is known that induced spoofing has the same signal structure as the authentic satellite signal. Therefore, the spoofing can generally be modeled as:

(3) $$x_{s}\left(t\right)=\sum_{i=1}^{N}\sqrt{P_{i}^{s}}D_{i}^{s}\left(t-\tau_{i}^{s}\right)C_{i}\left(t-\tau_{i}^{s}\right)\cos\left(2\pi \left(f_{0}+f_{d,i}^{s}\right)t+\phi_{i}^{s}\right),$$

where *N* is the number of satellites included in the spoofing, $P_{i}^{s}$ is the received power of *i*-th satellite, and $D_{i}^{s}\left(t\right)$ is the ±1-valued *i*-th signal’s data bit stream, $\tau_{i}^{s}$ is the *i*-th signal’s code phase, $f_{d,i}^{s}$ denotes the Doppler shift of the *i*-th authentic satellite signal in hertz, and $\phi_{i}^{s}$ is its initial carrier phase. For notational simplicity, the time indication of some parameters, e.g., $f_{d,i}^{s}$, have been omitted.

For the success of an attack, the spoofing will include most of the satellites in the authentic ones. For the *i*-th satellite signal, the core of GNSS signal processing is the correlation of the received signal with local replica:

(4) $$\ell_{i}\left(t,\tau_{i}\right)=C_{i}\left(t-{\hat{\tau }}_{i}^{a}\right)\cos\left(2\pi \left(f_{0}+{\hat{f}}_{d,i}^{a}\right)t+{\hat{\phi }}_{i}^{a}\right)$$

where ${\hat{\tau }}_{i}^{a}$, ${\hat{f}}_{d,i}^{a}$ and ${\hat{\phi }}_{i}^{a}$ are separately the estimated $\tau_{i}^{a}$, $f_{d,i}^{a}$, and $\phi_{i}^{a}$. The goal of a receiver’s tracking loops is to accurately drive the estimates ${\hat{\tau }}_{i}^{a}$, ${\hat{f}}_{d,i}^{a}$ and ${\hat{\phi }}_{i}^{a}$. It is well known that these parameters are estimated based on the outputs of correlators in tracking loops. However, when there is an induced spoofing, there will be a distortion on the correlator outputs. Therefore, the tracking loops cannot accurately obtain the estimations.

For the case of a GPS L1 C/A signal, through carrier wipe-off and coherent integration of one millisecond, the cross-correlation function of the local replica and the authentic signal can be written as:

(5) $$R\left(\tau^{\prime }\right)=\left\{\begin{array}{cc} 1-\left|\tau^{\prime }\right|, & \left|\tau^{\prime }\right|\leq 1\\ 0, & \mathrm{otherwise}, \end{array}\right.$$

where $\tau^{\prime }={\hat{\tau }}^{a}-\tau^{a}$ denotes the code phase lag in chips. It is noted that, for the sake of simplicity, the subscript *i* has been omitted.

At the beginning of induced spoofing, it is assumed that the spoofing lags behind the authentic signal $\Delta_{c}$ chips. Thus the corresponding cross-correlation of the induced spoofing with the local replica can be expressed as:

(6) $$R\left(\tau^{\prime }-\left(\Delta_{c}-\Delta_{r}t\right)\right)=\left\{\begin{array}{cc} 1-\left|\tau^{\prime }-\left(\Delta_{c}-\Delta_{r}t\right)\right|, & \left|\tau^{\prime }\right|\leq 1\\ 0, & \mathrm{otherwise}, \end{array}\right.$$

where $\Delta_{r}$ is the code rate difference between the induced spoofing and the authentic signal.

Therefore, the correlator output for prompt channel with neglected carrier phase error is shown below:

(7) $$P\left(\tau^{\prime }\right)=\sqrt{P^{a}}R\left(\tau^{\prime }\right)\mathrm{sinc}\left(\Delta f_{d}^{a}\right)+\sqrt{P^{s}}R\left(\tau^{\prime }-\left(\Delta_{c}-\Delta_{r}t\right)\right)\mathrm{sinc}\left(\Delta f_{d}^{s}\right)+\tilde{n},$$

where $\mathrm{sinc}\left(x\right)=\frac{\sin(\pi x)}{\pi x}$, $\Delta f_{d}^{a}={\hat{f}}_{d}^{a}-f_{d}^{a}$ and $\Delta f_{d}^{s}={\hat{f}}_{d}^{a}-f_{d}^{s}$ are the Doppler shift residuals of the authentic signal and the spoofing, respectively. The $\tilde{n}$ comes from AWGN and cross-correlation results between local replica and other satellite signals, which can be expressed as the following:

(8) $$\tilde{n}=\int_{0}^{1}x_{n}\ell_{i}\left(t,\tau_{i}\right)dt+\sum_{j\neq i}\int_{0}^{1}x_{a,j}\ell_{i}\left(t,\tau_{i}\right)dt+\sum_{j\neq i}\int_{0}^{1}x_{s,j}\ell_{i}\left(t,\tau_{i}\right)dt,$$

where $x_{a,j}$ and $x_{s,j}$ are separately the *j*-th satellite signal including in the authentic signal and spoofing.

It is assumed that the receiver has stably tracked the authentic signal before the induced spoofing attack. At the same time, for simplicity, it is assumed that the Doppler frequency of the spoofing signal is the same as that of authentic signal, which is called a “frequency lock” in reference [39]. Then the correlation value of the prompt channel is modeled as:

(9) $$P\left(\tau^{\prime }\right)=\sqrt{P^{a}}R\left(\tau^{\prime }\right)+\sqrt{P^{s}}R\left(\tau^{\prime }-\left(\Delta_{c}-\Delta_{r}t\right)\right)+\tilde{n}.$$

## 3. Detection of Induced Spoofing

### 3.1. The Proposed Method

In the correlation domain analysis of navigation signal quality, SCB is a common index to measure the navigation ranging error [37]. S-curve refers to the code-discriminatior curve of the early–late correlation value in the receiver code tracking loop, which varies with the different code-discriminator algorithms. The theoretical zero-crossing point of S-curve was located at the zero point of code tracking error. In fact, due to the influence of channel transmission distortion and nonlinear effect of the power amplifier, the discriminatior curve of code loop was usually locked in the place where a code phase deviation existed, resulting in SCB.

Assume that correlator spacing is *d*, the non-coherent power discriminator’s S-curve can be defined by the following formula [37]:

(10) $$S_{c}\left(\varepsilon \left(t\right),d\right)={\left|P\left(\varepsilon \left(t\right)-\frac{d}{2}\right)\right|}^{2}-{\left|P\left(\varepsilon \left(t\right)+\frac{d}{2}\right)\right|}^{2}$$

where $P\left(\cdot \right)$ is the correlation value between the received signal and local replica, the code phase difference between local replica and prompt channel is the argument of the correlation operator, and $\varepsilon \left(t\right)$, i.e., SCB, satisfied with the following formula:

(11) $$\varepsilon \left(t\right)=\arg\left\{S_{c}\left(\varepsilon \left(t\right),d\right)=0\right\}.$$

It should be noted that $\varepsilon \left(t\right)$ changes with time when there is induced spoofing.

According to Equations (8) and (9), we can get:

(12) $$\begin{array}{cc} S_{c}\left(\varepsilon \left(t\right),d\right) & ={\left|\sqrt{P^{a}}R\left(\varepsilon \left(t\right)-\frac{d}{2}\right)+\sqrt{P^{s}}R\left(\varepsilon \left(t\right)-\frac{d}{2}-\left(\Delta_{c}-\Delta_{r}t\right)\right)\right|}^{2}\\ & -{\left|\sqrt{P^{a}}R\left(\varepsilon \left(t\right)+\frac{d}{2}\right)+\sqrt{P^{s}}R\left(\varepsilon \left(t\right)+\frac{d}{2}-\left(\Delta_{c}-\Delta_{r}t\right)\right)\right|}^{2}, \end{array}$$

where the noise $\tilde{n}$ has been omitted.

Next, substituting Equations (5) and (6) into (12),

(13) $$\begin{array}{cc} S_{c}\left(\varepsilon \left(t\right),d\right)= & {\left|\sqrt{P^{a}}\left(1-\left|\varepsilon \left(t\right)-\frac{d}{2}\right|\right)+\sqrt{P^{s}}\left(1-\left|\varepsilon \left(t\right)-\frac{d}{2}-\left(\Delta_{c}-\Delta_{r}t\right)\right|\right)\right|}^{2}\\ & -{\left|\sqrt{P^{a}}\left(1-\left|\varepsilon \left(t\right)+\frac{d}{2}\right|\right)+\sqrt{P^{s}}\left(1-\left|\varepsilon \left(t\right)+\frac{d}{2}-\left(\Delta_{c}-\Delta_{r}t\right)\right|\right)\right|}^{2}. \end{array}$$

Combined with Equations (11) and (13), SCB can be obtained by solving the following equation:

(14) $$\begin{array}{c} {\left|\sqrt{P^{a}}\left(1-\left|\varepsilon \left(t\right)-\frac{d}{2}\right|\right)+\sqrt{P^{s}}\left(1-\left|\varepsilon \left(t\right)-\frac{d}{2}-\left(\Delta_{c}-\Delta_{r}t\right)\right|\right)\right|}^{2}\\ ={\left|\sqrt{P^{a}}\left(1-\left|\varepsilon \left(t\right)+\frac{d}{2}\right|\right)+\sqrt{P^{s}}\left(1-\left|\varepsilon \left(t\right)+\frac{d}{2}-\left(\Delta_{c}-\Delta_{r}t\right)\right|\right)\right|}^{2}. \end{array}$$

For convenience, we denote $A=\sqrt{P^{a}}$, $B=\sqrt{P^{s}}$, d=1. In addition, it is noted that $\Delta_{c}$ is the code phase difference in chips between authentic signal and spoofing at the beginning of the attack. As an example, letting $\Delta_{c}=-2$ will be consistent with the experiments in reference [39].

Then, since $R\left(\tau^{\prime }\right)$ is a piecewise function, the solution of Equation (14) needs a piecewise analysis. Therefore, the theoretical formula of SCB can be obtained as:

(15) $$\varepsilon \left(t\right)=\left\{\begin{array}{cc} 0, & 0<\Delta_{r}t\leq 0.5\\ \frac{B\left(\Delta_{r}t-0.5\right)}{2A-B-1}, & 0.5<\Delta_{r}t\leq \frac{A}{2B}\\ \frac{4B\left(2-\Delta_{r}t\right)-3A}{2\left(B-A\right)}, & \frac{A}{2B}<\Delta_{r}t\leq 1.5-\frac{A}{4B}\\ \frac{2B\left(2-\Delta_{r}t\right)}{A+B}, & 1.5-\frac{A}{4B}<\Delta_{r}t\leq 2.5+\frac{A}{4B}\\ \frac{8B\left(2-\Delta_{r}t\right)+3A}{2\left(4B-A\right)}, & 2.5+\frac{A}{4B}<\Delta_{r}t\leq 3.5\\ 2-\Delta_{r}t, & \Delta_{r}t>3.5. \end{array}\right.$$

On the other hand, when there is no induced spoofing, the theoretical formula of SCB can be obtained by solving the following equation:

(16) $$\left|A\left(1-\left|\varepsilon \left(t\right)-0.5\right|\right)\right|=\left|A\left(1-\left|\varepsilon \left(t\right)+0.5\right|\right)\right|.$$

Resulting in the SCB being:

(17) $$\varepsilon \left(t\right)=0.$$

**Figure 2:**
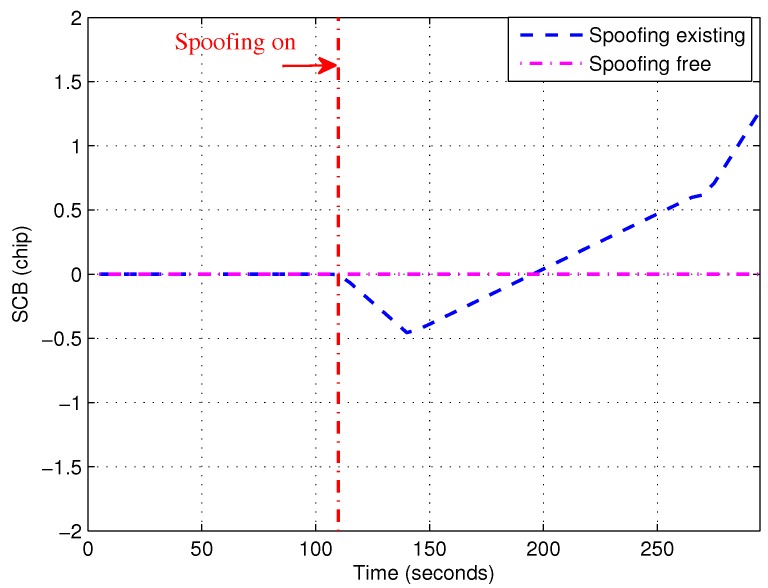
Theoretical S-curve-bias (SCB) curve with and without induced spoofing.

In order to gain an understanding about the theoretical SCB, Figure 2 shows the theoretical SCB with and without induced spoofing. As Equation (17) stated, SCB is always 0 when there is no spoofing. However, when there is an induced spoofing signal, SCB is changing over time which results in the gradual adjustment process. Specially, in Figure 2, SCB is reduced from 110 s to about 140 s. After that, SCB was increased from about 140 s. More importantly, the slope was almost unchanged from 110 s to about 140 s and from about 140 s to about 200 s. Thus there was slope mutation at about 140 s. Based on the above observation, the first-order derivative or the second-order derivative of SCB could be used to detect induced spoofing.

In this paper, a detection metric based on the derivative of SCB is proposed. In GNSS receivers, the first-order derivative of SCB can be computed using the finite differences method as:

(18) $$\delta_{t}=\frac{\varepsilon \left(t+\Delta t\right)-\varepsilon \left(t\right)}{\Delta t},$$

where $\varepsilon \left(t\right)$ is the SCB value at time *t*, Δt is the time difference between two adjacent timings. For a given threshold γ, if $\left|\delta_{t}\right|>\gamma$, it means that the first-order derivative exceeds the given threshold. Then the proposed algorithm declared an induced spoofing. Therefore, the proposed algorithm detected induced spoofing by utilizing the dynamic nature of gradual adjustment process. In experiments, SCB was calculated based on the method in reference [37]. In the code tracking loop of the software receiver, we can get the correlation values of the early code and the late code. After that, the S-type correlation curve can be obtained. Then SCB can be calculated based on the definition. More details can be found in the reference [37].

It is well known that there will be changes in the SCB when there is multipath. However, it is very unlikely that the changing process of multipath SCB is generally similar to that of induced spoofing due to the inherent randomness of multipath [40,41]. From this point of view, the proposed algorithm is not sensitive to multipath signals.

### 3.2. Probability Analysis

The detection of induced spoofing can be regarded as a binary detection problem, that is, to determine whether spoofing exists or not. Two hypotheses are defined as $H_{0}$ if an induced spoofing does not exist and $H_{1}$ if an induced spoofing does exist. Thus, given that (17) is the decision test, the hypothesis test can be expressed as:

(19) $$\left\{\begin{array}{c} H_{0}:\;\left|\delta_{t}\right|\leq \gamma ,\;\mathrm{without}\mathrm{spoofing}\\ H_{1}:\;\left|\delta_{t}\right|>\gamma ,\;\mathrm{with}\mathrm{spoofing}. \end{array}\right.$$

The probability of false alarm $P_{fa}$ is the probability that the hypothesis of the presence of an induced spoofing attack is accepted, but in fact, it is not present. The detection probability $P_{d}$ is the probability that the hypothesis of the presence of a spoofing attack is accepted, and it is present. For the calculation of $P_{fa}$ and $P_{d}$, we should obtain the distribution of SCB under the spoofing-present situation. However, according to the analysis above, for the induced spoofing, the inducing process has time-varying characteristics. In addition, the distribution of SCB depends on the tracking loop configuration of the victim receiver and the specific form of spoofing, i.e., changing speeds of code phase and power. But it is unknown to the receiver how code phase and power (carrier-to-noise ratio, $\frac{C}{N_{0}}$) of the spoofing will vary. Thus it is difficult for the victim receiver to predict the behavior of induced spoofing. It is also difficult to determine the specific distribution of SCB [37] when there is an induced spoofing. Under this circumstance, it is difficult to derive the analytical expression of the probability density function to compute $P_{fa}$ and $P_{d}$.

If the pattern of induced spoofing is given, $P_{fa}$ is also a function of the threshold value γ. Thus the probability of false alarm $P_{fa}$ can be calculated as the following:

(20) $$P_{fa}=\int_{\gamma }^{+\infty }p\left(T;H_{0}\right)dT,$$

where $T=|\delta_{t}|$, $p\left(T;H_{0}\right)$ denotes the probability density function of T when there is no induced spoofing.

Similarly, the detection probability $P_{d}$ can be calculated as the following:

(21) $$P_{d}=\int_{\gamma }^{+\infty }p\left(T;H_{1}\right)dT,$$

where $p\left(T;H_{1}\right)$ is the probability density function of T when there is an induced spoofing.

In experiments, as that in [3,34], the probability of false alarm $P_{fa}$ will be obtained when there is no induced spoofing as the following:

(22) $$P_{fa}=\frac{\#\left\{T>\gamma \right\}}{M},$$

where #{·} is the total number of satisfied argument conditions. $P_{fa}$ is explained as a relative frequency, that is a ratio between the number of times the test statistic exceeds the given threshold out of the total number of experiment realizations *M*.

When there is an induced spoofing, the detection probability $P_{d}$ will be obtained as the following:

(23) $$P_{d}=\frac{\#\left\{T>\gamma \right\}}{M}$$

$P_{d}$ is similarly explained as a relative frequency, that is a ratio between the number of times the test statistic exceeds the given threshold out of the total number of experiment realizations *M*.

For a given $P_{fa}$, the threshold is first calculated based on Equaiton (22). In addition, a similar hypothesis testing can be performed for the ratio method. In this paper, we calculated the detection probability every 10 s where the number 10 is selected as an experience value. That is to say, spoofing detection was performed within each small detection window. The performance of the proposed algorithm will be evaluated and compared with the ratio method.

## 4. Experiments

In this section, experiments are carried out to evaluate the performance of the proposed method. Based on the theoretical analysis of the previous section, the proposed induced spoofing detection method will be embedded into a conventional software GPS receiver which is developed in Civil Aviation University of China. The TEXBAT data sets [39] are used in all experiments.

### 4.1. Introduction of TEXBAT Data Sets

The radionavigation laboratory in the University of Texas at Austin produced the first public database (ds1–ds6) of signals affected by several types of spoofing attacks concerning GPS satellites in 2012. Two additional data sets, ds7 and ds8 were added to TEXBAT in August 2015. Therefore, there were eight different GPS L1 C/A spoofing data sets in TEXBAT [39,42], namely ds1–ds8, which represent different spoofing attack scenarios. They were based on two “clean data sets” replayed through the vector signal generator, and 25 Msps sampling rate data grabber attached in one case to a static antenna building on the campus of the University of Texas and in the other case to an antenna mounted on a vehicle, which travelled across the city [39].

In this paper, ds7 was chosen to evaluate the performance of the proposed method. In this scenario, it represented the so-called seamless takeover attack. There were no offsets in samples and time, and spoofing was already perfectly aligned since it was digitally added to the clean data sets. No obvious disruption can be observed in the tracking loop. The ds8 represents a zero-delay security code estimation and replay (SCER) attack, which was identical to the ds7, except for the spoofer guesses, and generated the navigation data bits in real time [42]. Since the proposed method did not employ security codes to detect spoofing attacks, its anti-spoofing performance in ds7 and ds8 will be the same. Therefore, ds8 was not be considered in the paper.

For ds7, spoofing is free from 0 to 110 s, data is identical to “clean static date sets” during this time. The spoofing signals are injected for each GPS L1 C/A signals at the 110-th second and the amplitude of spoofing varied nonlinearly. After that, the code phases of the spoofing relative to the counterpart authentic ones increased at a rate of 1.2 m per second, that is, $409.2\times 10^{-5}$ Hz. Finally, a 1.27μs clock offset is induced in the victim receiver which is described in the reference [42].

### 4.2. Ratio Test Detection Method

As a comparison, the intermediate spoofing detection algorithm based on the ratio test metric (called the ratio method) [33] is also used in this paper. As in [33], the detection metric of the ratio method is defined by the following formula:

(24) $$R_{d}=\frac{E_{i}^{\frac{d}{2}}+L_{i}^{\frac{d}{2}}}{\beta P_{i}}$$

where $E_{i}^{\frac{d}{2}}$, $L_{i}^{\frac{d}{2}}$ and $P_{i}$ represent the early, late and prompt correlator output over the in-phase branch, the superscript $\frac{d}{2}$ denotes the correlator spacing between the early/late correlator and prompt correlator, β is the correlation main peak slope. For more details, refer to [33]. According to the theory presented in [33], the threshold, e.g., $\gamma^{\prime }$, can be derived with a given false detection probability. Therefore, if $\left|R_{d}\right|>\gamma^{\prime }$, it is decided that the receiver has been attacked by a spoofing.

It is worth pointing out that the ratio method detects spoofing by finding the distortion of correlator output at a given timing. In other words, it detects spoofing based on the static distortions of the correlator output. In contrast, the proposed algorithm declares an induced spoofing attack by detecting the dynamic changes of the SCB.

### 4.3. Results of the Proposed Method

**Figure 3:**
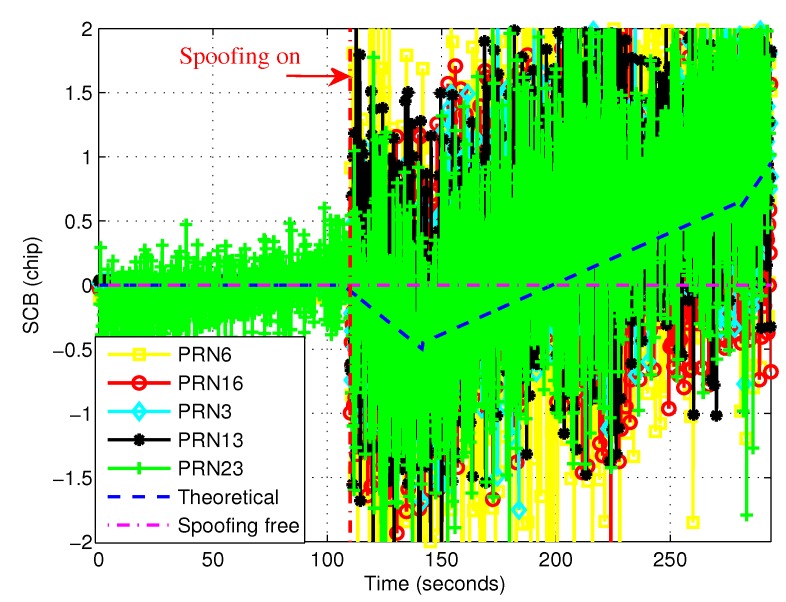
Comparisons between experimental SCB curve and theoretical SCB curve.

Figure 3 shows the comparisons between the experimental and theoretical SCBs. The time length was 294 s and the induced spoofing attack occurred at 110 s (vertical point-line). Each satellite actually had a theoretical SCB curve, but these theoretical curves coincided with each other and only one theoretical SCB curve is shown in Figure 3. For the experimental SCBs, five curves according to five tracked satellites are shown. From 0 to 110 s, there were almost no fluctuations, except for PRN23 which caused the other curves to be masked by the curve of PRN23. For PRN23, the fluctuation may have been due to poor signal quality or high noise conditions. It is noted that the experimental SCB curves take the fluctuations on both sides of the theoretical value. The main reason is that the theoretical SCB was derived by ommiting the noise $\tilde{n}$ in Equation (8). However, in fact, noise was unavoidable, which lead to fluctuations. On the other hand, the overall trend was consistent with the theoretical curve. In order to reduce the impact of fluctuations, the curves were filtered with a Butterworth digital filter. The coefficient vectors of the filter system function’s molecular polynomial and denominator polynomial are $b=[9.9419\times 10^{-4},2.0\times 10^{-4},9.9419\times 10^{-4}]$ and a=[1,−1.908,0.9218].

**Figure 4:**
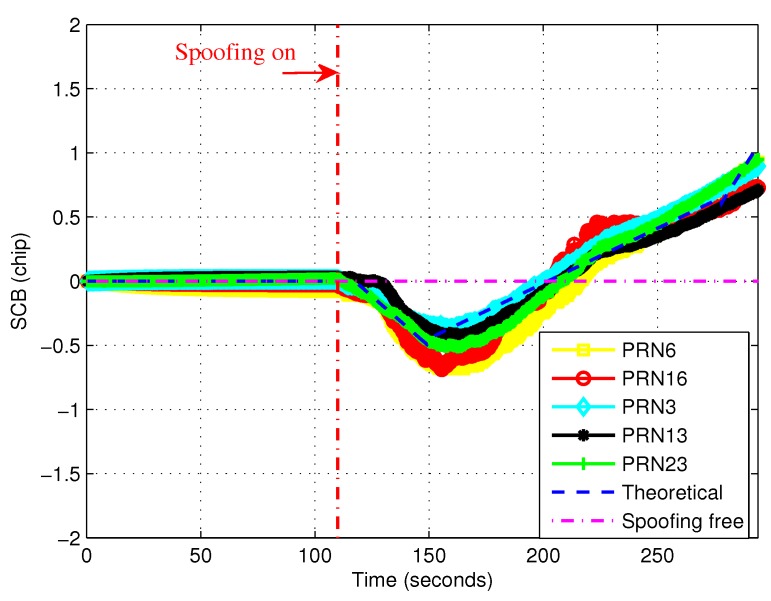
Comparisons between filtered experimental and theoretical SCB curves.

Figure 4 shows the filtered experimental SCB curves. It is more clear to show that the experimental curves were almost consistent with the theoretical curve. It is noted that experimental SCB values had a very small fluctuation around 0 when there was no spoofing in the first 110 s. From 110 s, the curve gradually deviated from zero. It indicates that there was an induced spoofing and it’s code phase lagged behind that of the authentic satellite signal. When the maximum negative value was reached, the value of SCB slowly increased to zero. At this time the spoofing was already very close (about within 0.5 chips) to the authentic signal. After arriving at zero point, the receiver tracking loop was controlled by induced spoofing. The tracking loop of the victim receiver locked on the induced spoofing and was slowly pulled out of the authentic signal. Then the value of SCB increases gradually.

**Figure 5:**
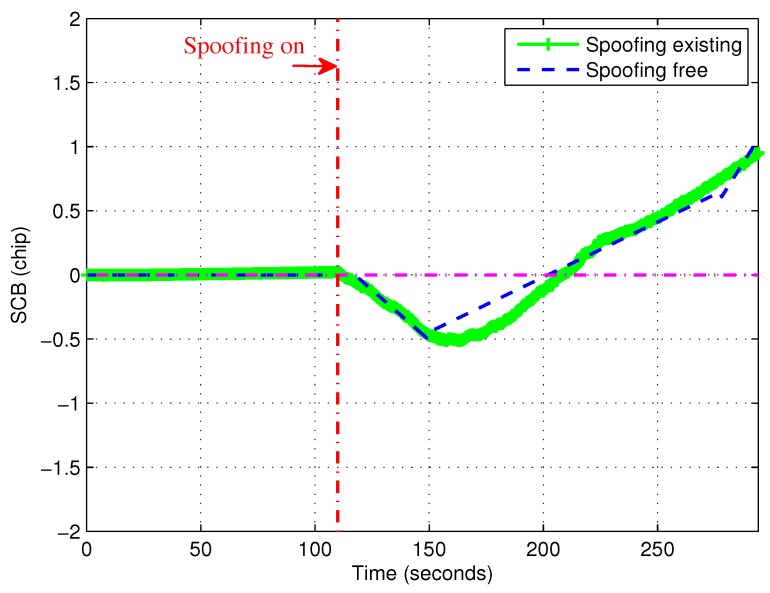
Experimental and the theoretical SCB curves for PRN 23.

In order to obtain more details, as an example, the SCB curve of PRN23 is shown in Figure 5. The experimental SCB curve had a good fit with the theoretical curve. As stated before, when the spoofing began to induce the victim receiver, the SCB curve changed gradually.

**Figure 6:**
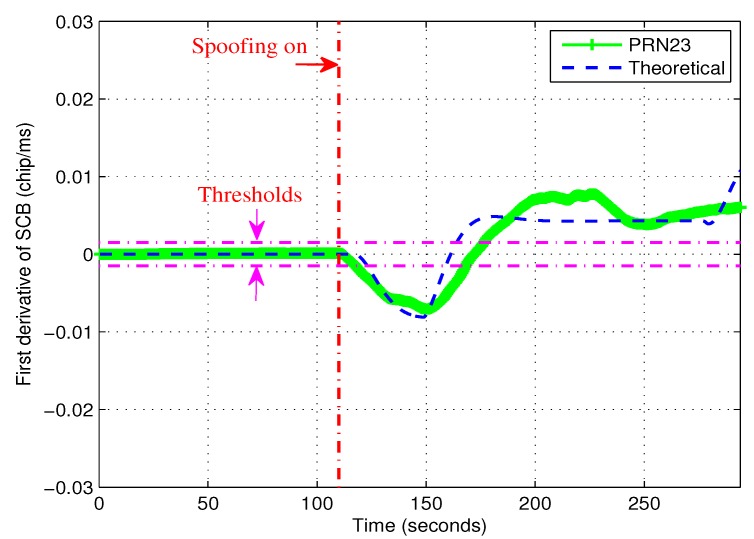
The first derivatives of experimental and the theoretical SCB curves for PRN 23.

For PRN23, the first derivative of SCB is shown in Figure 6. It is clear that the theoretical value with spoofing was significantly greater than that without spoofing. When there was spoofing, the experimental values fluctuated around the theoretical ones. The experimental values were close to zero before 110 s where there was no spoofing. More importantly, it was far less than the value with spoofing. Therefore, it was easy to find a reasonable detection threshold γ.

Table 1 summarizes the detection threshold γ corresponding to the false alarm probability $P_{fa}$ for the proposed algorithm. It is reminded that the thresholds are selected based on Equation (22).

### 4.4. Results of Ratio Method

**Figure 7:**
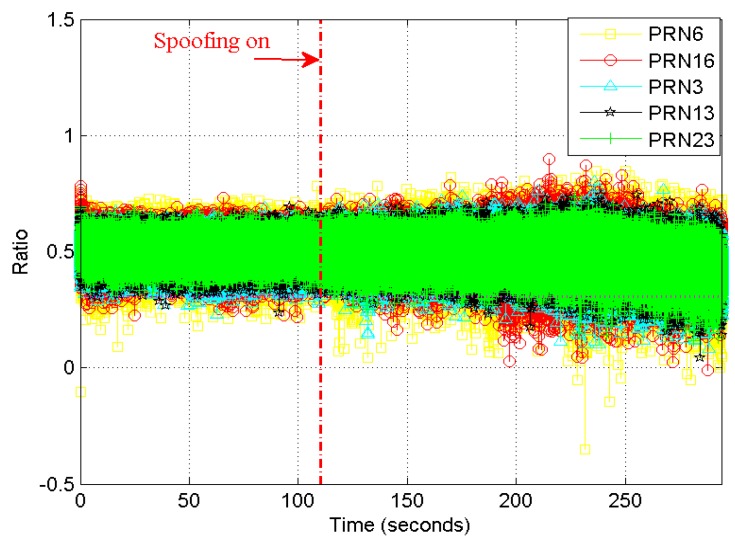
Ratio method curves during an induced spoofing attack.

For the ratio method, Figure 7 shows the curve during an induced spoofing attack. The time length was also 294 s. The induced spoofing was also added at the 110-th s. We can see that the changing of different satellites were almost the same. Before 200 s, the changing amplitude of the ratio method was not obvious. Thus it was difficult to detect an induced spoofing attack before 200 s. After that, the ratio method was slowly changing.

**Figure 8:**
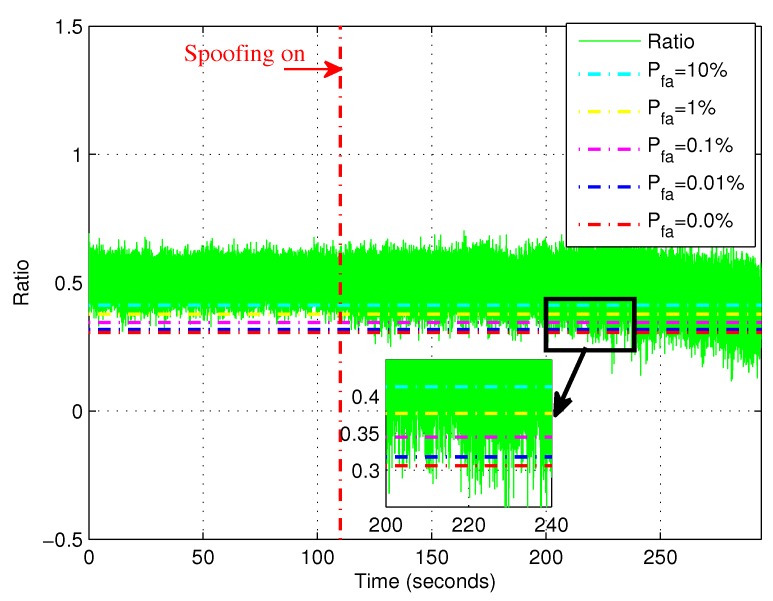
The ratio method curve for PRN 23. The solid curve is the ratio method metric. The dashed curves are detection thresholds according to different false alarm probabilities.

Figure 8 shows the ratio method curve of PRN 23. In Figure 8, the sold curve is the ratio test metric for PRN23. The different dashed curves are detection thresholds $\gamma^{\prime }$ according to different false alarm probabilities. The thresholds were increased with increasing of the false alarm probability. The values of detection threshold corresponding to different false alarm probability are summarized in Table 2.

### 4.5. Comparison of Two Methods

**Figure 9:**
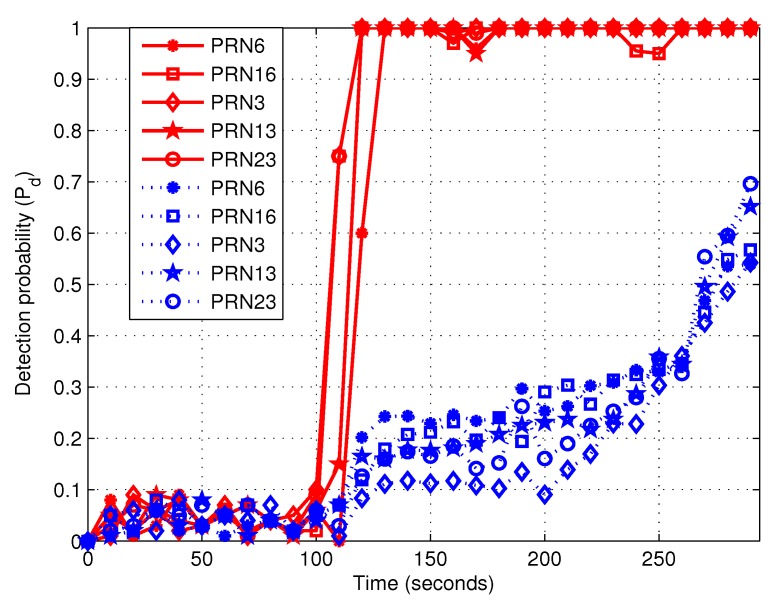
The relationship between detection probability and time length. The red solid and blue dashed curves are separately corresponding to the proposed algorithm and the ratio method. The false alarm probability was set to 0.1.

When the false alarm probability is set as 0.1, the thresholds of two algorithms are selected as those in Table 1 and Table 2. It is reminded that the detection probability is calculated every 10 s. Figure 9 shows detection probabilities with time for the proposed method and the ratio method, respectively. The red solid and blue dashed curves are separately corresponding to the proposed algorithm and the ratio method. It is noted that the solid and dashed curves are separately corresponding to the proposed algorithm and the ratio method. At about the 110-th s, the proposed method can detect spoofing and the detection probability is almost unity. However, until about the 294-th s, the ratio method reaches the maximal detection probability which is about 0.7. But it is still slightly lower than the proposed method. Therefore, compared with the ratio method, the proposed algorithm can detect induced spoofing at a much earlier stage, which is because the proposed method utilizes the dynamic feature of induced spoofing.

**Figure 10:**
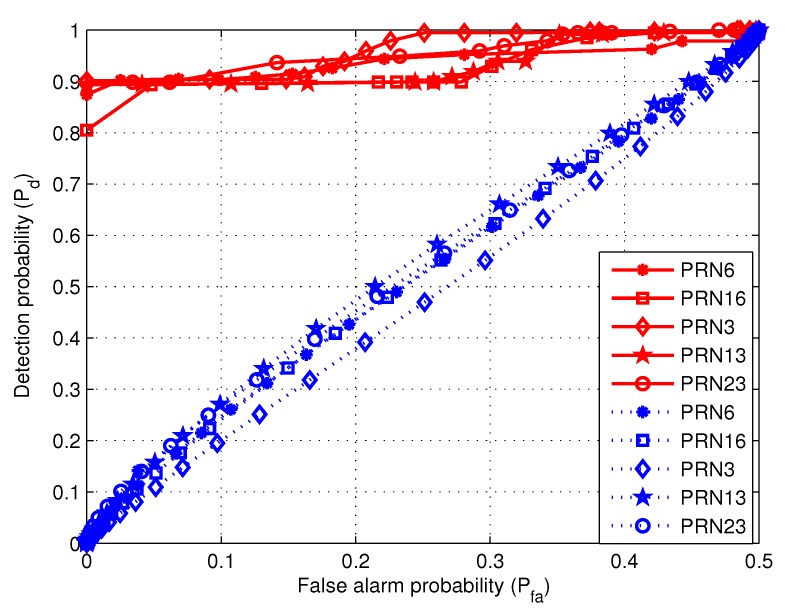
The relationship between detection probability and false alarm probability for the first 200 s data. The red solid and blue dashed curves are separately corresponding to the proposed algorithm and the ratio method.

**Figure 11:**
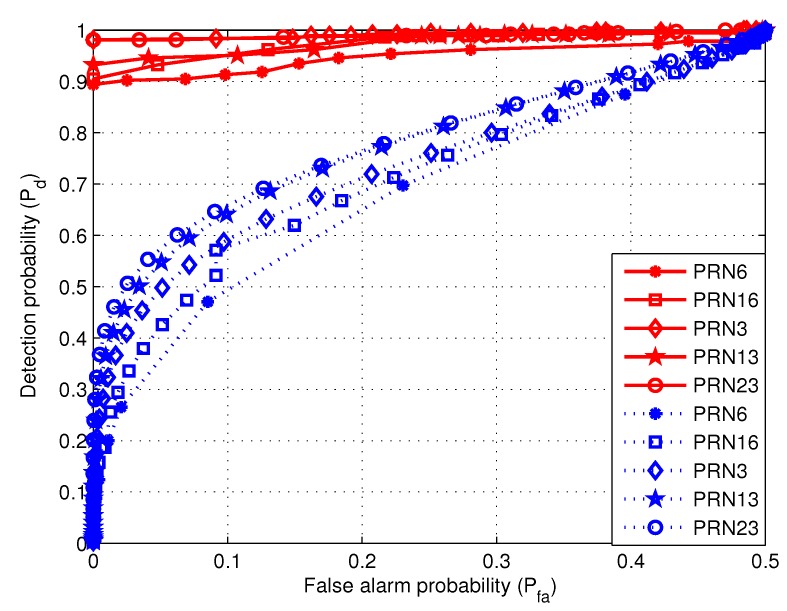
The relationship between detection probability and false alarm probability for the first 294 s data. The red solid and blue dashed curves are separately corresponding to the proposed algorithm and the ratio method.

In order to obtain more details, the detection probabilities of the two algorithms are compared for different lengths of the received signal. When the length of the data are separately 200 s and 294 s, Figure 10 and Figure 11 show the relationship between detection probability and false alarm probability.

Figure 10 shows the detection probabilities for the first 200 s data. As expected, $P_{d}$ tends to 1 for increasing $P_{fa}$ values. However, the curves corresponding to the proposed algorithm attain $P_{d}\to 1$ faster than the ratio method. Therefore, when the data length is 200 s, the proposed algorithm outperforms the ratio method.

When the data length is 294 s, the relationship between the detection probability and false alarm probability is shown in Figure 11. With much longer data length, the performance of the ratio method is significantly improved. It means that, for a given satisfied detection performance, the ratio method needs longer data. At the same time, the performance of the proposed algorithm is also improved. Moreover, the proposed algorithm performs still better than the ratio method.

## 5. Conclusions

In this paper, an induced spoofing detection algorithm is proposed. By gradually adjusting its parameters, the induced spoofing can capture victim tracking loops without creating loss of locks. However, it will lead to a significant change in the SCB. The proposed algorithm is based on the change of SCB to detect induced spoofing. In other words, the proposed algorithm utilized the dynamic feature of gradual adjustment process. More specifically, a detection metric based on the first derivative of SCB is defined in this paper. When the detection metric exceeds a given threshold, an induced spoofing will be declared. A series of experiments with the Texas Spoofing Test Battery (TEXBAT) are performed to verify the effectiveness of the proposed algorithm. The experimental results demonstrate that the proposed algorithm can detect induced spoofing in a earlier stage.

Future work includes deducing the theoretical threshold value for a given $P_{fa}$ or $P_{d}$. On the other hand, it is noted that the proposed algorithm detects induced spoofing based on the dynamic nature of gradual adjustment process. Then there is overlapping between the two correlation peaks corresponding to authentic signal and spoofing, respectively. When the induction process is finished, the correlation peak of spoofing will not be overlapped with that of authentic signal and the proposed algorithm may fail. Then the proposed algorithm could be combined with other techniques such as power level monitoring to detect spoofing. Therefore, another possible future direction could be combining the proposed algorithm with other detection methods.

## Figures and Tables

**Table 1:** The relationship between false alarm probability and detection threshold for the proposed algorithm.

	False Alarm Probability (%)	Detection Threshold
1	10	1.421 × $10^{-4}$
2	1	1.476 × $10^{-4}$
3	0.1	1.480 × $10^{-4}$
4	0.01	1.485 × $10^{-4}$
5	0	1.495 × $10^{-4}$

**Table 2:** The relationship between false alarm probability and detection threshold for the ratio method.

	False Alarm Probability (%)	Detection Threshold
1	10	0.455
2	1	0.413
3	0.1	0.379
4	0.01	0.342
5	0	0.315
